# Subclinical Thyrotoxicosis and Cardiovascular Risk: Assessment of Circulating Endothelial Progenitor Cells, Proangiogenic Cells, and Endothelial Function

**DOI:** 10.3389/fendo.2022.894093

**Published:** 2022-07-18

**Authors:** Jason Phowira, Sherin Bakhashab, Anuradha Doddaballapur, Jolanta U. Weaver

**Affiliations:** ^1^ Department of Endocrinology, Queen Elizabeth Hospital, Gateshead, Newcastle Upon Tyne, United Kingdom; ^2^ Translational and Clinical Research Institute, Newcastle University, Newcastle upon Tyne, United Kingdom; ^3^ Biochemistry Department, King Abdulaziz University, Jeddah, Saudi Arabia

**Keywords:** subclinical thyrotoxicosis, endothelial progenitor cells, cardiovascular risk, circulating angiogenic cells, proangiogenic cells, apoptosis

## Abstract

**Background:**

Subclinical thyrotoxicosis (SCT) is defined by low or undetectable thyroid-stimulating hormones and normal thyroid hormones. The treatment of SCT is uncertain despite being associated with increased cardiovascular risk (CVR) and mortality. Circulating endothelial progenitor cells (cEPCs) and circulating angiogenic cells (CACs) have been found to be reduced in conditions with CVR. We aimed to evaluate whether endothelial function and cEPC and CAC counts were reduced in SCT and to study the *in vitro* effect of triiodothyronine (T3) on proangiogenic cell (PAC) function from young healthy controls.

**Methods:**

cEPCs (quantified by flow cytometry, 20 SCT/20 controls), CACs following *in vitro* cultures (15 SCT/14 controls), paracrine function of CACs, endothelial function by flow-mediated dilation (FMD, 9 SCT/9 controls), and the effect of T3 on apoptosis and endothelial nitric oxide synthase (*eNOS*) expression in PACs were studied.

**Results:**

*p* < 0.001, CD133^+^/VEGFR-2^+^ 0.4 (0.0–0.7) vs. 0.6 (0.0–4.6), *p* = 0.009, CD34^+^/VEGFR-2^+^ 0.3 (0.0–1.0) vs. 0.7 (0.1–4.9), *p* = 0.002; while CAC count was similar. SCT predicted a lower cEPC count after adjustment for conventional CVR factors. FMD was lower in SCT subjects versus controls (% mean ± SD, 2.7 ± 2.3 vs. 6.1 ± 2.3, *p* = 0.005). *In vitro* studies showed T3 increased early apoptosis and reduced *eNOS* expression in PACs.

**Conclusions:**

In conclusion, SCT is associated with reduced cEPC count and FMD, confirming increased CVR in SCT. Future outcome trials are required to examine if treatment of this subclinical hyperactive state improves cardiovascular outcome.

**Clinical Trial Registration:**

http://www.controlled-trials.com/isrctn/, identifier ISRCTN70334066.

## Background

Endothelial dysfunction plays a pivotal role in the development of cardiovascular disease. Circulating endothelial progenitor cells (cEPCs) have been suggested to play an important role in endothelial regeneration. A lower number of cEPCs in peripheral blood, as defined by different combinations of surface markers CD34^+^, CD133^+^, and vascular endothelial growth factor (VEGFR)2^+^, or circulating angiogenic cells (CACs), previously known as early EPCs, has been associated with increased cardiovascular risk (CVR) (1–3).

CACs can be defined as CD3^+^CD31^+^CXCR4^+^ T cells during cultures of human peripheral blood mononuclear cells. CACs are required for colony formation and differentiation of early EPCs. They secrete high levels of angiogenic cytokines such as VEGF, interleukin-8, and matrix metalloproteinases (4).

Proangiogenic cells (PACs) refer to cells with hematopoietic surface markers that are responsible for neovascularization. Following injury and vascular remodeling, these cells are recruited to the sites of active angiogenesis to generate a proangiogenic environment and facilitate vascular repair (5). We have previously reported significantly lower levels of cEPCs and PACs in a model of subclinical cardiovascular disease in comparison to healthy controls, suggesting the roles of these cells as markers of vascular damage and increased CVR (6).

Early atherosclerosis, as defined by endothelial dysfunction, has been associated with the progression of carotid intima-media thickness and reduced cEPC number (7, 8). Furthermore, in conditions such as hyperlipidemia, subclinical hypothyroidism, diabetes mellitus, and sedentary lifestyle associated with either lower cEPCs or abnormal CACs and PACs, appropriate therapeutic interventions improved those abnormalities in cEPCs or PACs (9–12).

Subclinical thyrotoxicosis (SCT) is defined by low or undetectable serum thyroid-stimulating hormones (TSHs) and normal thyroid hormones, free thyroxine (FT4), and free triiodothyronine (FT3). SCT is a common disorder with an increased prevalence in older subjects; 1% between the ages of 60 and 80 years, increasing to 3% of individuals older than 80 years of age (13). SCT is associated with an increased prevalence of atrial fibrillation (14–16), and a few but not all studies have shown an association of SCT with increased mortality (15, 17). Moreover, a prospective cohort study revealed that the risk for atrial fibrillation and mortality from coronary heart disease in SCT patients was highest when the TSH level was below 0.1 mU/L (18). Among a population of SCT patients with a mean age of 66.5, Vadiveloo et al. reported a dose-dependent effect on the increased risk of cardiovascular morbidity and dysrhythmia with decreasing TSH measurement (19). Another study among community-dwelling women aged 65 and above also demonstrated that a prior history of hyperthyroidism was associated with a higher all-cause and cardiovascular mortality risk (20).

Therefore, given its potential contribution to the development of cardiovascular disease, we hypothesized that SCT was associated with an abnormal number of cEPCs or PACs, contributing to endothelial dysfunction and increased CVR. This study aimed to quantify cEPCs by flow cytometry and culture CACs from peripheral blood mononuclear cells in SCT in comparison to age- and sex-matched controls. Furthermore, we measured endothelial function, plasma asymmetric dimethylarginine levels, the *in vitro* paracrine function of CACs, and the effect of high T3 concentrations on apoptosis and eNOS expression of PACs.

## Methods

### Subjects

Twenty SCT (18 women) and 20 controls (matched for age, sex, and body mass index (BMI)) were included in this study (**Table 4**). SCT patients were recruited from the endocrine clinic (Queen Elizabeth Hospital, Gateshead) after confirming the diagnosis (abnormal TSH and normal FT4 and FT3, twice). SCT patients were observed irrespective of CVR factors and drugs, which could affect cEPCs/CACs, as both are very common in the elderly population. Therefore, we matched our patients and controls for sex, age, BMI, and risk factors for CVD.

Study subjects had their anthropometric measurements (height, weight, BMI), blood pressure, fasting lipid profile, asymmetric dimethylarginine (ADMA), and cEPC and PAC counts taken.

The control subjects were recruited from among friends and spouses of patients. We have included a few control subjects with CVR factors and matching medication to match SCT patients, providing the thyroid status of controls was normal. All subjects gave their written informed consent, and the local ethics committee approved the study (ethical approval (05/Q0901/104)). The authors had full access to the data and take responsibility for its integrity.

### Biochemical Measurements

FT4 (reference range, 9–25 pmol/L), FT3 (reference range, 3–6.8 pmol/L), and TSH (reference range, 0.4–4.0 mU/L) concentrations were measured by electrochemiluminescence immunoassay (Roche Diagnostics, Lewes, UK). Serum TC and HDL-C were assayed using automated enzymatic methods (Roche Diagnostics, UK). LDL-C was calculated using Friedewald’s equation. Plasma ADMA was measured by ELISA (DLD Diagnostika, Hamburg, Germany).

### Endothelial Function

The endothelial function of the brachial artery was performed by measurement of flow-mediated dilation (FMD) in 9 SCT and 9 controls using high-resolution ultrasonography, HDI 5000 system (Toshiba, Tokyo, Japan), and a 12-MHz linear transducer as previously described by our group (10, 21).

Briefly, the ultrasound system was connected to a personal computer equipped with a frame grabber and an artificial neural network wall tracking software (vessel image software, VIA). This software automatically tracks the anterior and posterior walls within a user-defined region of interest and also accommodates angulation of the artery up to 20° relative to the perpendicular. The diameter of the blood vessel was determined by averaging a large number of local vessel diameters. The B-mode images were processed at 25 frames per second. The vessel diameter, including the changes throughout the cardiac cycle, was displayed in real time, which allows ultrasound imaging to be optimized before starting the scan and transducer position to be adjusted for optimum tracking during the entire examination. The right brachial artery was scanned continuously for 2–10 cm above the elbow, with the participant lying supine after an initial 15-min rest.

After a baseline measurement for 2–4 min, transient vascular occlusion was obtained (5 min) with a blood pressure cuff inflated to 250 mm of mercury situated at the proximal forearm. Pulsed Doppler was used in assessing arterial flow at rest and immediately after tourniquet deflation. Brachial artery dilatation was recorded for 5 min after releasing the tourniquet. the FMD was automatically calculated by VIA as the ratio of mean diastolic vessel diameters 55 to 65 s after reactive hyperemia to the baseline diameter, expressed as a percentage change.

### Circulating EPC Quantification

cEPCs in peripheral venous blood (100 µl) were quantified within 4 h of blood collection by flow cytometry (LSRII, Beckton Dickinson, San Jose, CA, USA) after incubation in the dark with the following antibodies for 30 min: PE-Cy7 anti-CD14 (BD Pharmingen, San Jose, CA, USA), Per CP-Cy5.5 anti-CD 34 (BD Pharmingen, San Jose, CA, USA), APC anti-CD 133 (Miltenyi Biotec, Bergisch Gladbach, Germany), PE-anti-VEGFR-2 (KDR) (R&D Systems, Minneapolis, MN, USA), and FITC-anti-CD 144 (**Table 1**) (Alexis Biochemicals, San Diego, CA, USA). Isotype matching mouse anti-IgG was used as a control in Trucount tubes with beads (22). Cells were identified using BD FACS Diva™ software according to their forward and sideward scatter profiles in the lymphocyte gate after acquiring at least 100,000 events. The cEPC count was calculated using the software-calculated percentage after subtracting nonspecific background staining for positive events in the control tube. CD34^+^/VEGFR-2^+^, CD133^+^/VEGFR-2^+^ CD144^+^/CD34^+^, and CD144^+^/CD133^+^ cells were identified as cEPCs.

**Table 1:** Antibodies are used for the enumeration of cEPCs and progenitor cells.

Antibody	Fluorochrome	Volume
CD14	PE-Cy7	4 μl
CD34	PerCP-Cy5.5	5 μl
CD133	APC	5 μl
VEGFR2 (KDR)	PE	5 μl
CD144	FITC	10 μl

### Circulating Angiogenic Cells After Culturing Mononuclear Cells in SCT and Controls

CACs were the adherent mononuclear cells of a 4- to 7-day culture procedure (23). The number of CAC cells was assessed in 15 SCT and 14 control subjects. Mononuclear cells (1 × 10^6^) were plated on fibronectin-coated 24-well plates enriched with endothelial medium (basal medium with supplement pack, PromoCell, Heidelberg, Germany, supplemented with 20% fetal calf serum). The medium was changed after 72 and 120 h.

Counting was performed on day 5 in 15 randomly selected high-power fields (HPF, ×400) by fluorescence microscopy after incubation with acetylated-LDL (5 mg/L; Invitrogen, Carlsbad, CA, USA) and Ulex lectin (10 mg/L; Sigma, Saint Louis, MO, USA) for 4 h at 37°C. CAC cells were identified as cells with combined uptake of acetylated-LDL and Ulex lectin binding.

### VEGF Secretion From CACs

The supernatants were collected after centrifuging the conditioned media at 1,000×*g* for 20 min. VEGF measurement from the culture supernatant fluid of CAC cells was studied in 8 controls and 5 SCT subjects. This was done according to the manufacturer’s instructions (Human VEGF Immunoassay, R&D, MN, USA; Catalog No. DVE00). At the end of the cell culture, the cells were collected and counted to normalize VEGF concentration to 100 × 10^3^ CACs for patients and controls (24).

### Characterisation of PACs

PACs cultured from the group of healthy controls without CVD risk factors (additional control group) were grown in 6-well plates until ~75% confluent in endothelial medium (basal medium with supplement pack, Promocell, Heidelberg, Germany, supplemented with 20% fetal calf serum). To perform *in vitro* assays using T3, characterization of PACs was done to confirm PACs were early endothelial progenitor cells.

PACs were characterized for the expression of endothelial markers in comparison to mature endothelial cells, human umbilical vein endothelial cells (HUVECs), and macrophages. Peripheral blood mononuclear cells or HUVECs obtained from normal umbilical cords were cultured in 3 different media on fibronectin-coated 6-well plates.

The method used was quantitative real-time reverse-transcription PCR, otherwise known as qRT-PCR. RNA was extracted from the cells using the RNeasy Mini Kit (QIAGEN, Hilden, Germany) and reverse transcribed to produce cDNA using SuperScript^®^ VILO™ cDNA Synthesis Kit (Life Technologies, Paisley, UK). For all the cDNA preparations, 500 ng of RNA was used. The expected product size was around 100–200 bp. SYBR Green Master Mix (Life Technologies) was used, and once the samples were prepared, they were placed in the light cycler (StepOnePlus Real-Time PCR System, Life Technologies). Each sample contained 33 µl of fast SYBR Green Master Mix, 24.75 µl of d H_2_O, 0.875 µl of each of the primers, and 3.3 µl of cDNA. All the samples were run in triplicates.

The parameters for the run were 95°C for 10 min, followed by 40 cycles of 95°C for 15 s, annealing temperature ranging from 55 C to 60°C for 60 s, followed by 72°C extensions for 20 s. The comparative Ct method was used to calculate the relative expression of genes, which was normalized to the housekeeping gene TATA-box-binding protein (TBP). The primers’ sequence is summarized in **Table 2**.

**Table 2:** Primer sequences used for quantitative real-time RT-PCR.

Gene name and accession number		Primer sequence		Product size
1	*CD31*	Forward	AACAGTGTTGACATGAAGAGCC	148 bp
	NM_000442.4	Reverse	TGTAAAACAGCACGTCATCCTT	
2	*CD34*	Forward	GCGCTTTGCTTGCTGAGTTT	183 bp
	NM_001773.2	Reverse	GCCATGTTGAGACACAGGGT	
3	*CD144*	Forward	GATCAAGTCAAGCGTGAGTCG	114 bp
	NM_001795.3	Reverse	AGCCTCTCAATGGCGAACAC	
4	*VWF*	Forward	AGCCTTGTGAAACTGAAGCAT	154 bp
	NM_000552.3	Reverse	GGCCATCCCAGTCCATCTG	
5	*CD146*	Forward	TCCCGCAGCCCCTGAGAGAC	174 bp
	NM_006500.2	Reverse	CAGCGATAGCCGCCTCCTGC	
6	*CD309*	Forward	GCGGGCCAATGGAGGGGAAC	165 bp
	NM_002253.2	Reverse	AAGGCACCACGGCCAAGAGG	
7	*CD14*	Forward	CGGCGGTGTCAACCTAGAG	142 bp
	NM_001174105.1	Reverse	GCCTACCAGTAGCTGAGCAG	
8	*CD115*	Forward	TCCAAAACACGGGGACCTATC	133bp
	NM_005211.3	Reverse	TCCTCGAACACGACCACCT	
9	*TRα-1*	Forward	GCTGCAGGCTGTGCTGCTA	179 bp
	NM_199334.3	Reverse	CGATCATGCGGAGGTCAGT	
10	*eNOS*	Forward	AGGCTTTTGATCCCCGGGTCC	164 bp
	NM_000603.4	Reverse	GTTGTAGGGGCCGGACATCTCCA	
11	*TBP*	Forward	GCCACGCCAGCTTCGGAGAG	184 bp
	NM_003194.4	Reverse	TCAGTGCCGTGGTTCGTGGC	

### PACs and Thyroid Hormone Receptor Studies

We studied if PACs express thyroid hormone receptor *TRα-1* by assessing *TRα-1* mRNA levels performed by KAPA2G Robust HotStart ReadyMix (Kapa Biosystems, Wilmington, MA, USA) RT-PCR (**Table 2**). The size of DNA fragments was determined by comparison with a 100-bp DNA ladder molecular weight marker (Promega, Fisher Scientific, Waltham, MA, USA).

### T3 Incubation Studies

PACs cultured from healthy controls were grown in 6-well plates until ~75% confluent. The medium was then removed and washed gently with 1× phosphate-buffered saline. All cells were incubated for 48 h with varying concentrations of T3 in serum-free media (1× and 2× physiological T3 concentrations). The physiological concentration of T3 was defined as 0.003 ng/ml (25).

### Apoptosis Assay

Apoptosis in PACs was assessed using BD Annexin V PE apoptosis detection kit I, according to the manufacturer’s specifications. Translocation of phosphatidylserine (PS) from the inner to the outer leaflet of the plasma membrane occurs during the early stages of apoptosis. Annexin V is a Ca^2+^-dependent phospholipid-binding protein that has a high affinity for PS and, hence, allows for the identification of cells undergoing early apoptosis. 7-Amino-actinomycin (7-AAD) serves as a viability probe during flow cytometry to exclude end-stage apoptotic and dead populations of cells. Viable cells with intact membranes exclude 7-AAD, whereas the membranes of dead and damaged cells are permeable to 7-AAD. Early apoptosic cells are Annexin V-PE positive and 7-AAD negative, and cells that are in late apoptosis or already dead are PE Annexin V-PE and 7-AAD positive. One time, T3 concentration was used as a standard.

### Quantification of eNOS

For quantification of *eNOS* transcripts, real-time reverse transcriptase PCR was performed on Step One Plus Real-Time PCR system (Life Technologies). PAC cultures were harvested after a 48-h T3 treatment, and total RNA was extracted using the Trizol (Invitrogen) method. First-strand cDNA at a concentration of 20 ng/µl was synthesized according to the manufacturer’s protocol (Invitrogen). PCRs were assayed in triplicates with a reaction mix containing 1× Fast SYBR Green Master Mix (Applied Biosystems), 20 ng of cDNA, and 0.125 µM of gene-specific primers (**Table 2**) . The parameters for the *eNOS* run were 95°C for 10 min, followed by 40 cycles of 95°C for 15 s, 58°C for 60 s, and 72°C for 20 s; those for *TBP* were 95°C for 10 min, followed by 40 cycles of 95°C for 15 s, 60°C for 60 s, and 72°C for 30 s. Negative controls (water) were run to ensure there was no contamination. Melting curve analysis was performed at the end of 40 cycles, and products were run on an agarose gel to ensure the specificity of the amplified product. The comparative Ct method was used to calculate the relative expression of *eNOS*, which was normalized to the reference gene *TBP*. The sequence of the primers is shown in **Table 2**.

### Statistical Analysis

The data were analyzed using the SPSS-15 statistical package (Chicago, IL, USA). The normality of the samples was assessed by the Shapiro–Wilk test. The difference between groups was analyzed using the Student’s *t*-test or by the Mann–Whitney *U* test (for not normally distributed data). The correlation between cEPCs/CACs and other parameters was calculated by Spearman’s correlation analysis. Linear regression analysis was carried out using cEPCs and CACs (after log transformation) as dependent variables after adjusting for age, BP, BMI, lipids, and the patient group as categorical variables. The values are provided in mean ± SD or median (range), except for the *in vitro* experiments, which were reported as mean ± SEM. Corrected *p*-values have been reported for significant results (two-tailed significance, <0.05).

## Results

### Clinical Study

Patients with SCT and controls were well matched for age, BMI, and cardiovascular risk factors. Patients with SCT and controls were well matched for age, BMI, CVR factors, and drugs involved in CVR. The following medications were taken by SCT patients: 6 aspirin, 8 antihypertensives (including 4 ACE inhibitors), 5 antidiabetic agents, and 7 cholesterol lowering. This was matched by medications taken by controls (4 aspirin, 7 antihypertensives (including 4 ACE inhibitors), 6 antidiabetic agents, 6 cholesterol lowering (*p* = NS)). Serum TSH was lower, as expected, in SCT compared to controls: median (range); 0.1 (<0.01–0.36) vs. 2.3 (1.0–4.0), *p* < 0.001 (**Table 3**).

**Table 3:** Summary of differences in parameters in two groups.

Characteristics	Controls	SCT	*p*-value
Number	20	20	NS
Age (years)	62 (10.4)	61 (12.7)	0.87
Females	18	18	NS
BMI (kg/m²)	26.6 (6.8)	27.7 (6.2)	0.60
TSH (mU/L)	2.32 (1.00–4.00)	0.1 (<0.01–0.36)	<0.001
FT4 (pmol/L)	15.5 (1.8)	16.6 (3.0)	0.32
FT3 (pmol/L)	5.4 (0.6)	5.6 (0.9)	0.30
Systolic BP (mmHg)	131.8 (18.9)	133.7 (14.1)	0.73
Diabetes	3	3	NS
Diastolic BP (mmHg)	80.1 (8.5)	81.2 (10.4)	0.74
TC (mmol/L)	5.2 (1.1)	4.8 (0.9)	0.36
LDL-C (mmol/L)	3.0 (0.9)	3.0 (0.9)	0.58
HDL-C (mmol/L)	1.6 (0.5)	1.4 (0.3)	0.14
ADMA (µmol/L)	0.8 (0.2)	1.0 (0.4)	0.08
FMD (%)	6.1 (2.1)	2.7 (2.3)	0.005

NS, Non-significant.

Data are expressed as mean (SD) or median (range). *SCT*, subclinical thyrotoxicosis; *TSH*, thyroid-stimulating hormone; *FT4*, free thyroid hormone; *FT3*, free triiodothyronine; *BP*, blood pressure; *TC*, total cholesterol; *LDL-C*, low-density lipoprotein-cholesterol; *HDL-C*, high-density lipoprotein cholesterol; *ADMA*, asymmetric dimethyl arginine; *FMD*, flow-mediated dilatation. To convert to metric units, FT4 (pmol/L to ng/dl, divide by 12.87), TC, HDL-C, and LDL-C (mmol/L to mg/dl, multiply by 38.6). ^a^Observation was done in 11 SCT and 9 control subjects. ^b^Observation was done in 9 SCT and controls.

cEPCs were significantly reduced in SCT compared to controls (**Table 4**). However, there was no significant difference in CAC cell number after tissue culture between the groups.

**Table 4:** cEPCs at the lymphocyte gate and CAC cell numbers after culture in SCT and controls.

Cell population (%)	Controls	SCT	*p*-value
CD34^+^	2.3 (0.6–2.7)	1.1 (0.2–2.1)	<0.001
CD133^+^	1.9 (0.2–4.7)	0.9 (0.0–2.3)	0.01
CD34^+^/CD133^+^	1.2 (0.4–3.2)	0.6 (0.1–1.5)	0.17
CD133^+^/VEGFR-2^+^	0.6 (0.0–4.6)	0.4 (0.0–0.7)	0.009
CD34^+^/VEGFR-2^+^	0.7 (0.1–4.9)	0.3 (0.0–1.0)	0.002
CD34^+^/CD144^+^	0.7 (0.3–4.8)	0.4 (0.1–1.1)	0.002
CD144^+^/CD133^+^	0.6 (0.2–4.4)	0.4 (0.1–0.8)	0.005
CACs after culture (cells/15HPF)^a^	34 (8–54)	46 (17–72)	0.07

HPF, high-power field. ^a^CAC culture was done in 14 controls and 15 SCT subjects.

There was a significant positive relationship between cEPCs and serum TSH (r/p): CD34^+^, 0.4/0.009; CD133^+^/VEGFR-2^+^, 0.3/0.04; and CD34^+^/VEGFR-2^+^, 0.4/0.004. Similar positive relationship was found between FMD and selected cEPCs/CACs (r/p): CD34^+^, 0.5/0.03; CD34^+^/VEGFR-2^+^, 0.7/0.003; CACs, 0.85/0.004; and CD144^+^/CD34^+^, 0.3/0.03. Similar positive relationship was found between CEPCs and FMD (Spearman’s, r/p): CD34^+^, 0.5/0.03; CD34^+^/VEGFR-2^+^, 0.7/0.003; CD144^+^/CD34^+^, 0.6/0.01; and CD144^+^/CD133^+^, 0.5/0.03.

In multiple regression analyses, SCT significantly predicted lower cEPC number after adjusting for age, BMI, BP, CVRs (smoking status, IHD, diabetes, use of aspirin/angiotensin-converting enzyme inhibitor/angiotensin receptor blocker intake, total cholesterol (TC), high-density lipoprotein (HDL-C), low-density lipoprotein (LDL-C), standardized β coefficient/*p*-value), CD34^+^ (−0.68/0.001), CD133^+^ (−0.51/0.016), CD133^+^/VEGFR-2^+^ (−0.59/0.006), CD34^+^/VEGFR-2^+^ (−0.58/0.012), CD144^+^/CD34^+^ (−0.618/0.001), and CD144^+^/CD133^+^ (−0.617/0.001).

### Endothelial Function

FMD was lower in SCT subjects studied compared to controls; 9 in each group (% mean ± SD, 2.7 ± 2.3 vs. 6.1 ± 2.3, *p* = 0.005).

### Asymmetric Dimethylarginine Measurements

There was a trend for higher plasma ADMA levels (studied in 9 HC and 11 SCT) in SCT (µmol/L mean ± SD, 1.0 ± 0.4 vs. 0.8 ± 0.2, *p* = 0.08) but not statistically significant (**Table 3**).

### Paracrine Function of CACs

The secretion of VEGF by CACs was similar between SCT and controls (mean ± SD, pg/ml/100 × 10^3^ CACs; controls, 82.9 ± 90.9; SCT, 77.8 ± 57.7) (24).

### *In Vitro* Studies

#### Characterisation of PACs in a Model of SCT

**Figure 1:**
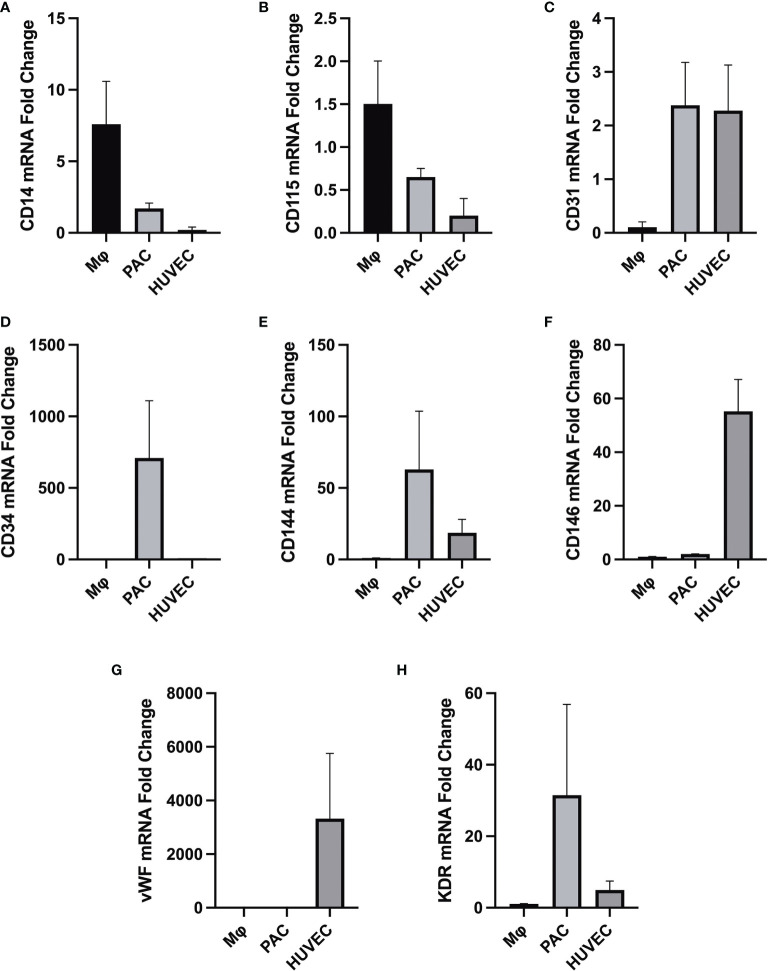
Differential gene expression (analyzed by RT-qPCR) in macrophages (MФ), PACs, and human umbilical vein endothelial cells (HUVEC), all isolated from the same donor (*n* = 3). Gene expression of macrophage-specific markers **(A)** CD14 and **(B)** CD115. **(C)** Gene expression profile of endothelial cell surface marker CD31. **(D)** Gene expression of the hematopoietic stem cell surface marker CD34 in PACs. **(E)** CD144 (VE-cadherin) is expressed in endothelial cells but not in macrophages. **(F)** CD146 (melanoma cell adhesion molecule) cell adhesion molecule used as a marker for mature endothelial cell lineage. **(G)** Gene expression of endothelial cell-specific marker vWF, upregulated in mature endothelial cells but not in PACs and macrophages. **(H)** KDR vascular endothelial growth factor receptor 2 (VEGFR-2), which is highly expressed in endothelial cells HUVECs and PACs. HUVECs serve as a control cell type for comparison. All data were normalized to the reference gene TATA-box-binding protein (TBP). It appears that PACs are distinctively different from macrophages given their characteristic gene signature.

We confirmed that PACs are appropriate endothelial cells to study the vascular effect of T3 as they express endothelial markers (**Figure 1**).

**Figure 2:**
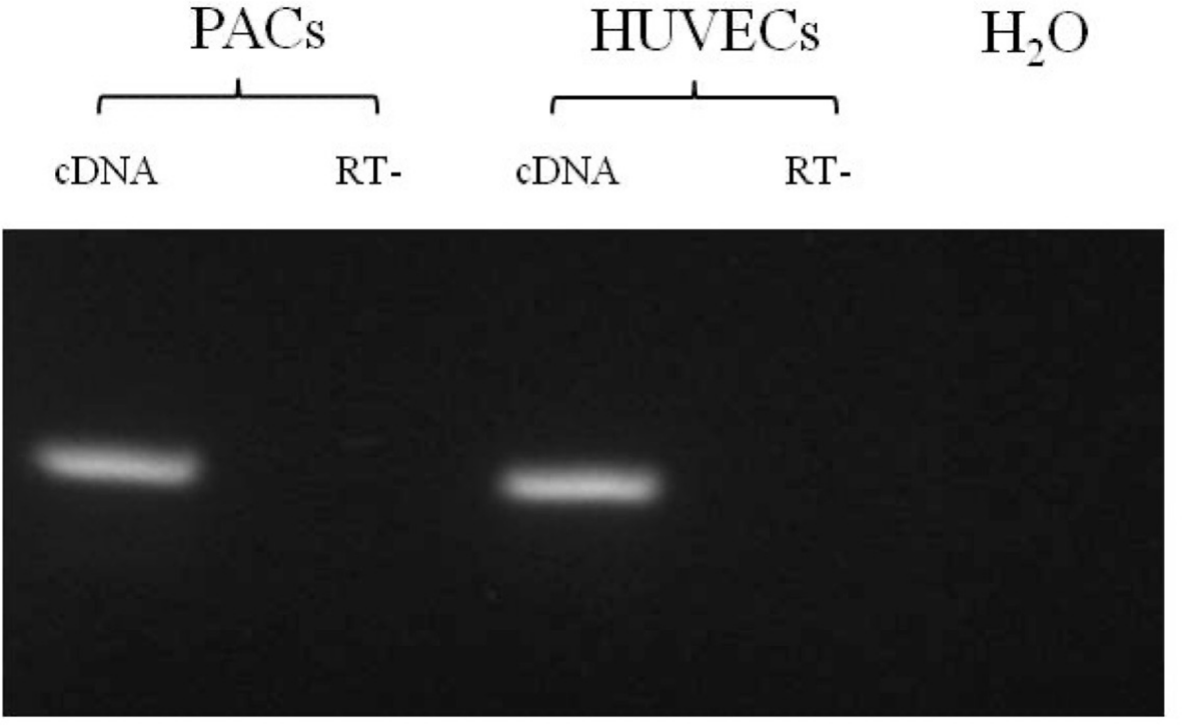
RT-PCR for *TRα1* in PAC and HUVEC samples. All samples are shown positive for *TRα1* mRNA expression. RT−, without reverse transcriptase. The size of DNA fragments was determined by comparison with a 100-bp DNA ladder molecular weight marker.

We confirmed that the PACs express thyroid receptor *TRα1*, as shown in **Figure 2**.

#### Apoptosis

**Figure 3:**
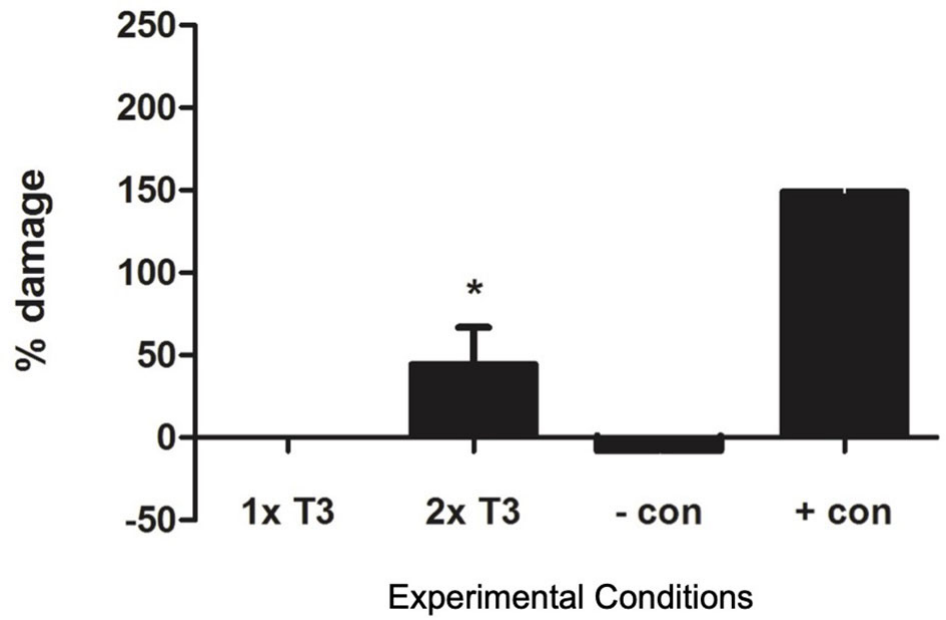
Graph showing apoptosis in proangiogenic cells (PACs) after staining with Annexin V and 7-AAD. Data are represented as mean ± SEM. ^*^ *p* < 0.05 versus normal (1× T3) and *n* = 11. +con, positive control, −con, negative control.

We found that *in vitro* thyrotoxic conditions generated by 2× physiological T3 concentrations led to a significant increase in the percentage of damaged PACs due to apoptosis (*n* = 11, *p* < 0.05, **Figure 3**).

#### Quantification of *eNOS*

**Figure 4:**
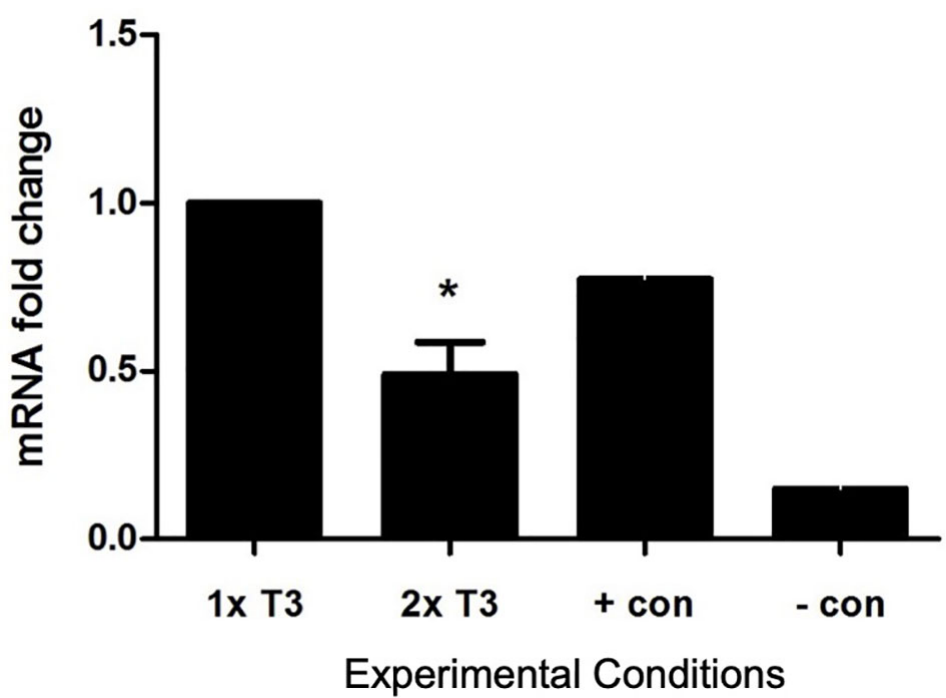
Effect of triiodothyronine (T3) on the expression of *eNOS* in PACs. Gene expression was quantified by real-time PCR and normalized to *TBP* (reference gene TATA-box-binding protein). Data are represented as mean ± SEM. ^*^ *p* < 0.05 versus normal (1× T3). +con, positive control; −con, negative control.

The addition of 2× normal concentrations of T3 to *in vitro* cultures of PACs reduced *eNOS* expression by 51% which was statistically significant (*p* < 0.05) (**Figure 4**).

## Discussion

We report, for the first time to our knowledge, that SCT is associated with a lower cEPC count and reduced FMD. Various subtypes of cEPCs were studied together with the mechanism behind endothelial dysfunction in SCT.

We studied SCT subjects in the presence of cardiovascular risk factors due to the preponderance of SCT in this group of subjects. The cEPC count in SCT was still significantly lower than in controls after adjusting for risk factors, suggesting that lower cEPCs were associated with SCT and therefore could contribute to increased vascular risk in this condition. Given that we studied middle-aged elderly subjects with CVD risk factors, we were unable to grow PACs in tissue cultures from those patients to study their function in detail. Therefore, we only studied CACs and cEPCs from those patients, while PAC cultures have been established from healthy individuals to study the model of subclinical thyrotoxicosis.

### Circulating EPCs

cEPCs are a heterogeneous population of cells characterized by the expression of surface antigens CD34^+^, VEGFR-2^+^, and/or CD133^+^ identified by fluorochrome-labelled antibodies and fluorescence-activated cell (FACS) analysis to discriminate peripheral blood cell subsets.

It appears that no single definition of cardiovascular progenitor cells has been agreed upon, and it is unknown which is the best antigenic profile or ratio of surface antigens to identify progenitor cells linked to CVD risk (26). The circulating endothelial progenitor cells (EPCs) are the most studied vascular progenitor cells (27). It is a general view that triple marking with CD34, CD133, and KDR is the most stringent and rigorous criterion for flow cytometry (27).

CD34 is an adhesion molecule expressed on hematopoietic stem cells, vascular progenitors, and certain microvascular endothelium, which identifies the progenitor cell population most closely and inversely linked to CVD risk (26). In our study, CD34^+^ cells were most significantly reduced in SCT versus controls. The SCT state independently predicted the reduction of cEPCs after controlling for CVD risk, confirming that the SCT state itself could be classed as increased CVD risk. This confirms previous studies showing an association between SCT and cardiovascular morbidity and mortality.

Vascular endothelial growth factor receptor type 2 (KDR) is generally considered suitable for demonstrating the endothelial commitment of progenitor cells, being expressed at a more immature stage than CD31 and vWF (28). In our study, CD34+VEGFR+2 cells were significantly reduced in SCT versus controls.

CD133 is a surface antigen with an unknown function that identifies more immature progenitors and has been proposed as the most appropriate marker for EPCs because it is not expressed on mature endothelium (29). However, CD133^+^ cells have been demonstrated to predict CV events to a lesser degree than other phenotypes (2). This may be the reason why there was no difference in CD34^+^/CD133^+^ between SCT and controls.

The circulating EPCs, defined as CD34^+^KDR^+^ cells, are reduced in the presence of classical risk factors for CVD, such as diabetes, smoking, hypercholesterolemia, and hypertension (1, 26, 30), as well as in the context of the three common manifestations of established coronary artery disease (CAD), cerebrovascular disease, and peripheral artery disease (PVD) (1, 31–33). Furthermore, among healthy subjects, a negative correlation has been reported between progenitor cells and endothelial function. Consistently, CD34^+^KDR^+^ cell count correlates with cumulative indexes of CV risk (1) and is an independent predictor of atherosclerosis progression and CV events (2, 34).

### Circulating Angiogenic Cells and Paracrine Function

We found that CAC count and VEGF secretion after culture were similar in both groups studied. This may be related to the small number of cell cultures carried out.

We hypothesize that the following factors could contribute to lower cEPCs in SCT, foremost being reduced nitric oxide (NO) bioavailability as NO has been shown to be the principal factor for the recruitment of cEPCs (35). Unfortunately, there are no studies on plasma NO levels in either SCT or overt thyrotoxicosis. Other humoral factors such as VEGF, cardiac abnormalities (atrial fibrillation, heart failure, ventricular dysfunction), increased oxidative stress, procoagulant state, and other proinflammatory factors may contribute to lower cEPCs in SCT.

### *In Vitro* Model of SCT

We have shown in this *in vitro* study for the first time expression of thyroid hormone receptors in PACs, suggesting a role of thyroid hormones in their function. We used an *in vitro* model of T3 excess as an approximation to an *in vivo* model of SCT. We have found a reduced eNOS expression in PACs in higher T3 concentrations, suggesting that higher tissue T3 levels affect eNOS expression in PACs. Furthermore, we showed that an increased concentration of T3 increased apoptosis of PACs, which could possibly explain the mechanism for lowering cEPCs. Although one is unable to make direct comparisons between *in vivo* cEPCs and *in vitro* cultured PACs, we have shown comprehensively that PACs demonstrate features of early endothelial cells, characterized by a significantly different molecular signature from cultured macrophages and HUVECs. PACs appear to clearly express endothelial markers such as CD144, thus making it a suitable model for studying endothelial function.

### Nitric Oxide Availability in SCT

We studied plasma ADMA in a small group of subjects to predict NO availability. Higher plasma ADMA and lower l-arginine/ADMA ratio (suggesting low NO availability) have been reported in overt thyrotoxicosis (36, 37). However, there had been no reports on plasma ADMA levels in SCT. In our study, ADMA levels were higher in SCT compared to the control group, but not statistically significant. The lack of significant difference may be related to the small number studied.

### Endothelial Function in SCT

The key mediator of endothelial function and the most important vasodilatory substance produced by the endothelium is nitric oxide (NO). It was originally identified as an endothelium-derived relaxing factor. Constitutive activity of endothelial NO synthase (eNOS) is responsible for the production of endothelial NO, maintaining relaxation of smooth muscles, thus leading to vasodilation. Reduced eNOS production resulted in decreased NO production, which was associated with endothelial dysfunction *via* reduced smooth muscle response. No direct experiments are available on how SCT may affect cEPCs; however, it is known that thyroid states affect bone marrow function, in particular myeloid cell lineages. It has been shown that SCT affects platelet volume associated with CVR (38).

Aicher et al. documented the essential role of endothelial nitric oxide synthase in the mobilization of stem and progenitor cells (35). This crucial information is in keeping with findings from our *in vitro* model of SCT, confirming that NO is involved in endothelial dysfunction and cEPC mobilization from the bone marrow.

This is the first study, to our knowledge, reporting reduced endothelial function in SCT. In our study, FMD was significantly lower in SCT compared to controls when assessed in 9 subjects in each group after correcting for age differences. Previous studies assessing FMD in SCT have not shown consistent results. One study reported lower FMD in SCT, but the result was not statistically significant (39). In another study, a significant reduction of FMD was reported after SCT was iatrogenically induced using thyroxine in 22 multinodular goiter subjects (from 10.7% ± 2.7% to 5.4% ± 1.7% (*p* < 0.001) after suppressing TSH to less than 0.5 mU/L (40).

### Management of SCT

We have just reported that treatment of SCT can lead to a 100% cure using radioiodine therapy (41). However, there are no randomized clinical studies to guide us on the ideal management of SCT or the reduction of cardiovascular risk. Moreover, at present, despite several recommendations established by various professional associations, there are no clear guidelines to aid healthcare professionals. The latest guideline by NICE suggests that SCT should be managed in adults with (1) two TSH readings <0.1 mlU/L at least 3 months apart and (2) evidence of thyroid disease (positive thyroid antibodies or a goiter) or symptoms of thyrotoxicosis. Measuring TSH levels routinely every 6 months should be considered in all adults with untreated SCT, with further evaluation of FT4 and FT3 if the TSH level is not within the reference range (42). A brief summary of the guidelines for the management of SCT has been published in our previous paper (41). The Medical Research Council-funded pilot study in the United Kingdom showed difficulty with the recruitment of patients with SCT into intervention with radioiodine therapy. Furthermore, according to the most comprehensive meta-analysis including large database analysis, individuals with subclinical hyperthyroidism demonstrate a 41% increase in relative mortality from all causes versus euthyroid control subjects (43). The related commentary suggested that patients with SCT should be treated to reduce cardiovascular risk (44).

Thus, the association of lower cEPCs and FMD in SCT further substantiates the necessity of treating this condition to lower the increased cardiovascular risk. However, as this is a new finding in a relatively small number of subjects, our results will need to be confirmed in future randomized controlled trials in a large number of SCT subjects before and after achieving a euthyroid state.

## Conclusion

Patients with SCT display poor vascular health *in vivo* and *in vitro*, which is consistent with increased cardiovascular risk. Randomized controlled interventional trials are indicated to explore if that increased risk can be reversed.

## Data Availability Statement

The original contributions presented in the study are included in the article/supplementary material. Further inquiries can be directed to the corresponding author.

## Ethics Statement

The studies involving human participants were reviewed and approved by Gateshead and South Tyneside Local Research Ethics committee on 13.12.2006 (05/Q0901/104). The patients/participants provided their written informed consent to participate in this study.

## Author Contributions

JW designed the experiment. JW and AD performed the experiment and data analysis. JP, SB, and AD drafted the main text, figures, and tables. JW supervised the work and reviewed the text. JW contributed to funding acquisition. All authors have read and agreed to the published version of the manuscript.

## Funding

This research was funded by Gateshead NHS Research and Development Fund, Queen Elizabeth Hospital Charitable Fund.

## Conflict of Interest

The authors declare that the research was conducted in the absence of any commercial or financial relationships that could be construed as a potential conflict of interest.

## Publisher’s Note

All claims expressed in this article are solely those of the authors and do not necessarily represent those of their affiliated organizations, or those of the publisher, the editors and the reviewers. Any product that may be evaluated in this article, or claim that may be made by its manufacturer, is not guaranteed or endorsed by the publisher.

## Abbreviations

cEPCs, circulating endothelial progenitor cells; PACs, proangiogenic cells; CACs, circulating angiogenic cells; CVR, cardiovascular risk; SCT, subclinical thyrotoxicosis; TSHs, thyroid-stimulating hormones; FT4, free thyroxine; FT3, free triiodothyronine; TBP, TATA-box-binding protein; PS, phosphatidylserine; 7-AAD, 7-amino-actinomycin; BP, blood pressure; TC, total cholesterol; LDL-C, low-density lipoprotein-cholesterol; HDL-C, high-density lipoprotein cholesterol; ADMA, asymmetric dimethyl arginine; FMD, flow-mediated dilatation; VEGF, vascular endothelial growth factor; HUVECs, human umbilical vein endothelial cells; PMBCs, peripheral blood mononuclear cells.
